# Future Perspectives on the Automation and Biocompatibility of Molecularly Imprinted Polymers for Healthcare Applications

**DOI:** 10.1021/acs.macromol.4c01621

**Published:** 2025-02-01

**Authors:** Saweta Garg, Pankaj Singla, Sarbjeet Kaur, Francesco Canfarotta, Eirini Velliou, James A. Dawson, Nikil Kapur, Nicholas J. Warren, Shoba Amarnath, Marloes Peeters

**Affiliations:** † University of Manchester, School of Engineering, Engineering A Building, Booth East Street, Manchester, M13 9QS, United Kingdom; ‡ Newcastle University, Newcastle upon Tyne, Tyne and Wear, NE1 7RU, United Kingdom; § MIP Discovery, Colworth Park, Sharnbrook, MK44 1LQ, Bedfordshire, United Kingdom; ∥ University College London, Centre for 3D Models of Health and Disease, Charles Bell House, London, W1W 7TY, United Kingdom; ⊥ University of Leeds, School of Mechanical Engineering, Woodhouse Lane, Leeds, LS2 9JT, United Kingdom; # School of Chemical, Materials and Biological Engineering, University of Sheffield, Sir Robert Hadfield Building, Mappin Street, Sheffield, S1 3JD, United Kingdom

## Abstract

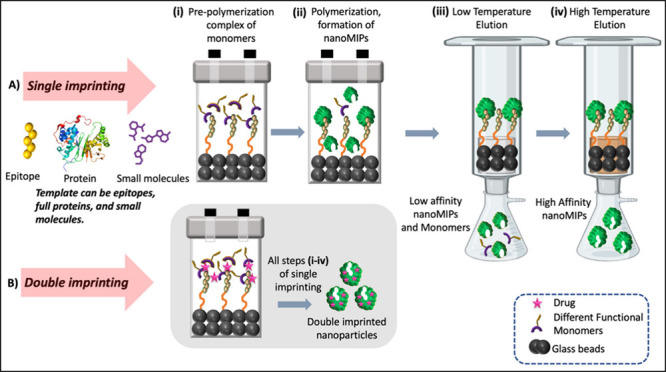

Molecular recognition is of crucial importance in several healthcare applications, such as sensing, drug delivery, and therapeutics. Molecularly imprinted polymers (MIPs) present an interesting alternative to biological receptors (e.g., antibodies, enzymes) for this purpose since synthetic receptors overcome the limited robustness, flexibility, high-cost, and potential for inhibition that comes with natural recognition elements. However, off the shelf MIP products remain limited, which is likely due to the lack of a scalable production approach that can manufacture these materials in high yields and narrow and defined size distributions to have full control over their properties. In this Perspective, we will confer how breakthroughs in the automation of MIP design, manufacturing, and evaluation of performance will accelerate the (commercial) implementation of MIPs in healthcare technology. In addition, we will discuss how prediction of the *in vivo* behavior of MIPs with animal-free technologies (e.g., 3D tissue models) will be critical to assess their clinical potential.

## 1 Introduction

Molecular recognition is of crucial importance for several scientific applications, including separation, catalysis, sensing, and drug delivery.^1,2^ However, natural recognition elements such as antibodies and enzymes possess limited stability and flexibility in use, in addition to having high cost and potential for inhibition.^3^ Therefore, researchers are continuously searching for synthetic substitutes that overcome these drawbacks. Molecular imprinting is one of the leading technologies to develop biomimetics, which is based on the creation of specific cavities in a 3D polymeric network that are complementary to the spatial configuration and chemical functionality of the chosen template molecule (i.e., the target).^4^ A unique property of these Molecularly Imprinted Polymers (MIPs) is the ability to tailor these materials to virtually any target of interest, ranging from ions to small organic molecules, to proteins and even large entities such as whole cells and bacteria.^5^ Moreover, MIPs represent a versatile, scalable, and cost-effective approach for the manufacturing of synthetic receptors, which can exhibit similar or superior affinity to commercial antibodies.^6^ Due to their enhanced robustness, ability to customize the material to the chosen application, and straightforward production process, MIPs have found application in several areas of healthcare including medical diagnostics. For instance, MIPs have been researched for the early diagnosis of cancer via detecting specific biomarkers with electrochemical and surface plasmon resonance-based sensors.^7−9^ In addition, imprinting is an animal-free technology, which is pivotal because since the existence of recombinant technologies, nearly 1 million animals have been used (and potentially sacrificed) in Europe for the production of antibodies used in diagnostics.^10^

The first scientific mention of molecular imprinting was nearly a century ago when Polyakov reported in 1931 that when silica gels were made in the presence of another molecule, the resulting polymers would selectively absorb that specific compound.^11^ In 1949, Pauling presented experiments by Dickey which demonstrated that silica gels had been prepared by “procedures analogous to the formation of antibodies.^12^ However, due to the limited stability and reproducibility of these silica materials, there was not much interest in the technology until the groups of Wulff and Klotz independently presented the first examples of molecular imprinting in the 1970s in synthetic organic polymers.^13,14^ The introduction of a general noncovalent approach by the group of Mosbach in the early 1980’s significantly extended the use of monomers and broadened the scope of the technology.^15^ The most cited research work remains a report of the group by Mosbach in *Nature* in 1993, which demonstrated that MIPs could have selectivity comparable to biological receptors.^16^ Since then, there has been an exponential increase in the number of studies reporting on MIPs.

Generally, MIPs can be manufactured to form various architectures such as membranes, layers, microparticles, or nanoparticles. Traditional MIP synthesis involved the production of (heterogeneous) microparticles, which suffer from low affinity, template leaching, and slow binding kinetics.^17^ However, due to their low-cost and enhanced robustness, these MIPs have found commercial applications for purification and separation where capacity is more important than sensitivity.^18^ More recently, the advances in nanotechnology have enabled the production of uniform nanoMIPs), which can rival the binding affinity of antibodies.^19^ In particular, these nanosystems are water-soluble, have a much higher surface-to-volume ratio, and exhibit enhanced binding kinetics. This provides an exciting opportunity to explore the use of these materials for healthcare applications, such as sensing, drug delivery, and nanomedicine. However, despite their seemingly simplistic production processes, off-the-shelf MIP products remain limited. A reason for this could be that MIPs to date have mostly been considered as “antibody replacements”, such as in their use as pseudo-immunoasays.^20,21^ While MIP-based assays exist with lower cost, enhanced robustness and a significantly better limit of detection compared to commercial tests, such as in the case of SARS-CoV-2,^22^ it is challenging to convince industry to move away from antibodies and legislative barriers around adopting new assay modalities can pose significant issues. However, we argue that MIPs can possess multiple functionalities that go beyond synthetic recognition: dual imprinting approaches and further modification of MIPs with, for instance, enzymes have provided the opportunity to explore drug delivery and theragnostic applications. For these applications, it is crucial to obtain materials with a high yield and narrow and defined size distribution to have full control over nanoMIP properties. In this Perspective, we will mention which breakthroughs are needed in the coming years to accelerate the (commercial) implementation of MIPs in healthcare technology. This will involve discussions around automating MIP development and assessment of the *in vitro* and *in vivo* behavior of these nanomaterials.

## 2 Automating MIP Development and Manufacturing

**Figure 1:**
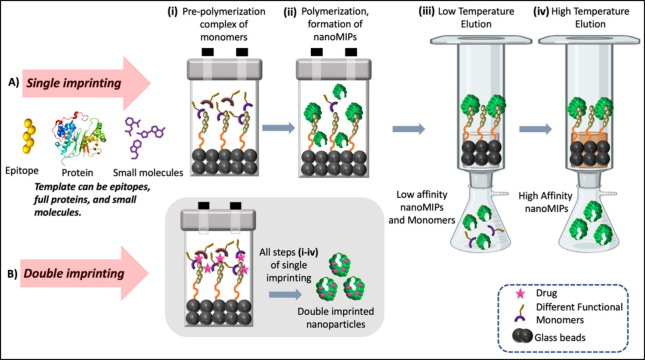
Solid phase synthesis of A) single imprinted and B) double imprinted nanoparticles for different templates, for example, small molecules, proteins, and larger macromolecules such as virus particles.

There are several approaches to producing molecularly imprinted nanoparticles (nanoMIPs). A popular method is the so-called solid-phase approach, where the solid-phase is used as an affinity medium to produce nanoMIPs with uniform high affinity binding characteristics.^23^ A general synthesis protocol for this method has been reported by Canfarotta et al.^24^ In short, this involves attaching the template, or in the case of a larger macromolecule a representative epitope, to functionalized glass beads. After introduction of the monomer mixture and subsequent polymerization, a series of elution steps at different temperatures is employed to collect homogeneous nanoMIPs with high affinity for the target, which is feasible due to the use of thermoresponsive monomers. Figure 1A summarizes this solid-phase approach for small scale production in a flask in the lab, and Figure 1B highlights an innovative double imprinting approach where two templates are introduced at the stage of the prepolymerization complex.

Most receptors and biomarkers of pharmaceutical interest are proteins. In principle, it is feasible to perform imprinting with the whole protein, and this has been attempted for several proteins (e.g., lysozyme, trypsin) that are low-cost when purchased commercially in quantities that are required for imprinting (∼1 mg).^25,26^ However, the majority of proteins are high-cost due to their complicated production process and sophisticated conformational structure that highly depends on the environment (pH, temperature, salts, use of buffers). Epitope imprinting, where a specific protein region is used for imprinting, is a popular approach that overcomes the aforementioned issues.^26,27^

While the solid-phase approach for nanoMIP production is promising, it has several distinct drawbacks. At the moment, there is no automated protocol for selection of monomers to provide materials with optimum affinity. Thus, most papers use a combination of the monomers described by Canfarotta et al.,^24^ covering a range of noncovalent interactions such as hydrogen bonding, ionic interactions, and hydrophobic interactions. This can be supplemented with monomers with fluorescent or redox capabilities to aid sensing.^28^

Computational modeling holds promise in this regard, and artificial intelligence and machine learning (ML) approaches are currently revolutionizing how we design and develop materials for a vast array of technological applications.^29,30^ In the context of nanoMIPs, data-driven optimization and sensing are becoming crucial for the efficient generation of nanoMIPs with excellent sensitivity and selectivity.^31^ Novel developments such as artificial intelligence (AI) and machine learning (ML) have not yet been extensively explored for MIPs, most likely due to the lack of high throughput production. ML can be used to predict imprinted polymer functionality before carrying out experiments by determining the optimal interactions between the target template and the functional monomer interactions.^32,33^ It can also be used to optimize various factors affecting synthesis and sensor performance, including monomer concentration, cross-linkers, initiators, and reaction temperature and media, for a range of applications. For example, Dykstra et al. developed a data-driven framework based on the synthesis and sensing performance of MIPs for cortisol detection with 72 sets of synthesis parameters with replicates.^34^ Based on the established framework, the synthesis parameters were optimized and validated experimentally, leading to a significant 1.5-fold increase in sensitivity. Yarahmadi et al. used ML based on an array of nonlinear regression algorithms to predict the imprinting factor of various MIPs.^35^ Using experimental data sets and inputs, including pH, template, monomer, solvent, the distribution coefficient of the MIP and the distribution coefficient of the nonimprinted polymer, the most important factors in influencing the imprinting factor were determined. Such approaches can dramatically reduce the number of experimental trials required and are therefore expected to be critical in the future design and application of nanoMIPs.

Moreover, it is crucial to have a scaled-up approach in place that can produce particles with precisely defined size and molecular weight in high yields. There are no commercial reactors available for MIP manufacturing yet, and literature reports on reactor designs are sparse; thus, synthesis is therefore mostly restricted to the use of standard laboratory flasks. The first automatic reactor for the synthesis of nanoMIPs was introduced by Poma et al., in 2013, who used an iniferter-type initiator to control polymerization and enabled recycling of the template via elution of the nanoMIPs rather than the solid-phase with immobilized template.^23^ This reactor was updated to facilitate production of nanoMIPs for proteins, which require mild (aqueous) conditions, which was achieved using ammonium persulfate (APS) and tetramethylethylenediamine as initiators at room temperature.^23^ However, these reactors are not widely implemented in the community due to their limited yields, cumbersome separation process, and lack of precise control over polymer formation, since it is not possible to monitor polymerization conditions *in situ.* A logical step would thus be to explore flow systems or automated polymerization platforms. The first automated reactor preparing MIP macroparticles was readily reported by Zourob et al., in 2006, using mineral oil or perfluorocarbon as continuous phase to form the particles in one-step continuous flow.^36^ However, the use of a solid-phase leads to complications due to posing diffusion barriers and difficulty to disperse the immobilized glass beads in the reactor. Moreover, scaling-up processes might lead to hot spots forming in the reactor, which can have serious safety implications. Automated platforms that enable self-optimization for identifying the best performing materials have shown promise in this respect.^37,38^ These platforms can develop models that enable hybrid *in silico* and experimental screenings of the polymer parameter space and monitor a range of important polymerization parameters (e.g., molecular weight, size, temperature, pH) *in situ*(39,40) and with careful reactor design can handle multiphasic and rheologically complex systems while maintaining good control over reaction conditions.^41^ While the size of nanoMIPs is conventionally determined via dynamic light scattering or electron microscopy, molecular weight is not typically recorded in literature reports, yet this could support assessment of the degree of homogeneity of the system. Monitoring the evolution of molecular weight of nanoMIPs will provide a better fundamental understanding of their production process and how target and functional monomers interact. The latter is particularly important for larger macromolecules that exhibit multiple binding sites, where it is often not clear how many functional monomers are involved in selective recognition. Thus, adapting these automated reactor systems used in polymer chemistry to MIP synthesis is expected to lead to breakthroughs in the (large scale) manufacturing of synthetic receptors; experience of automated systems inherently gives highly controlled and repeatable reaction outcomes, which in turn can underpin Good Manufacturing Practice, as required.

However, while innovations in reactor engineering enable us to have precise control over polymer formation, none of these parameters are directly linked to the affinity of the material. Therefore, it will be critical to combine novel reactor systems with high throughput screening approaches. While there are methods (such as via modeling, isothermal titration calorimetry or nuclear magnetic resonance) in place to screen the prepolymerization complex, which is a measure of affinity, it is not commonplace to have high throughput approaches in place after MIP production.^29,42^ With emerging advances in reactor engineering and a significant reduction in MIP production time, we believe that this will be an important area of focus. High throughput surface plasmon resonance (SPR) is an example of a technique that can facilitate high throughput screening in a 96-well plate format; while this is not an equipment that is standardly available in laboratories, it is appealing because it does not require labeling of the nanoMIPs to achieve detection.^43^ A more common alternative would be to consider pseudo-ELISA type assays or array formatted systems for electrochemical detection. This is possible since nanoMIPs typically contain ample functional groups, such as amine and carboxylic acid moieties, making it straightforward to modify them postpolymerization with suitable probe molecules. The alternative is to embed functional monomers with an integrated fluorescent or redox (e.g., ferrocene) functionality in the monomer mixture.^44^ However, fluorescent probes are typically bulky, and it must be carefully considered how well they are integrated in the overall polymer structure (due to having different reactivity ratios) and what their influence is on overall affinity.^45^ Therefore, computational approaches for monomer screening should also involve the inclusion of probe molecules to assess their impact on binding.

## 3 Predicting and Assessing nanoMIP Performance

### 3.1 Biochemical Assays

There are increased reports of the use of nanoMIPs for healthcare applications, but these nanomaterials have not yet been tested in clinical trials. Most studies assess the biocompatibility (IUPAC definition: Ability to be in contact with a living system without producing an adverse effect) of nanoMIPs through *in vitro* functional assays using either immortalized cell lines or primary cells which assess their binding phenotype, cytotoxicity and proliferative effect.^45^ The general conclusion of these reports tends to be that “nanoMIPs have the potential to replace biological therapeutics”. So while the application of nanoMIPs in healthcare is an exciting field, our understanding of the *in vivo* interactions of nanoMIPs remains underdeveloped due to lack of standardized testing protocols and evidence reported in the literature, especially with regard to their biodistribution, cytotoxicity, and clearance. It is worth noting that these properties are highly dependent on the surface chemistry and size of the resulting nanomaterial, its dose, mechanical properties, and method of administration. Moreover, considering the cross-linked nature of the nanoMIPs, determining the stability of the nanosystem *in vivo* and the impact of potential degradation products will be key. As such, it will be required to analyze each nanoMIP formulation individually to determine and predict its behavior *in vitro* as well as *in vivo.*

A study by Haupt’s group that evaluated the cytocompatibility of MIPs on human keratinocytes and axillary-hosted bacteria demonstrated that MIPs do not perturb the skin flora or lead to skin irritation, which was assessed via quantifying the amount of pro-inflammatory cytokines produced in addition to standard cytotoxicity experiments. Therefore, this presents a first step toward using these nanoMIPs for cosmetic or pharmaceutical formulations for skincare applications.^46^ In 2010, the group of Shea was one of the first to report on the use of imprinted materials for therapeutic function and application via considering their impact on systemic distribution.^47^ NanoMIPs, composed of acrylamide- and acrylic-acid-based monomers (dose = 30 mg/kg), were injected intravenously into immunocompetent mice. Over a period of 2 weeks, there was no significant difference in body weight between control mice or those who were administered nanoparticles (NPs), suggesting no apparent cytotoxic effects. Fluorescent images of the histological sections of the mice liver demonstrated that the nanoMIPs were concentrated in the liver, which gives an indication of their method of clearance.

**Figure 2:**
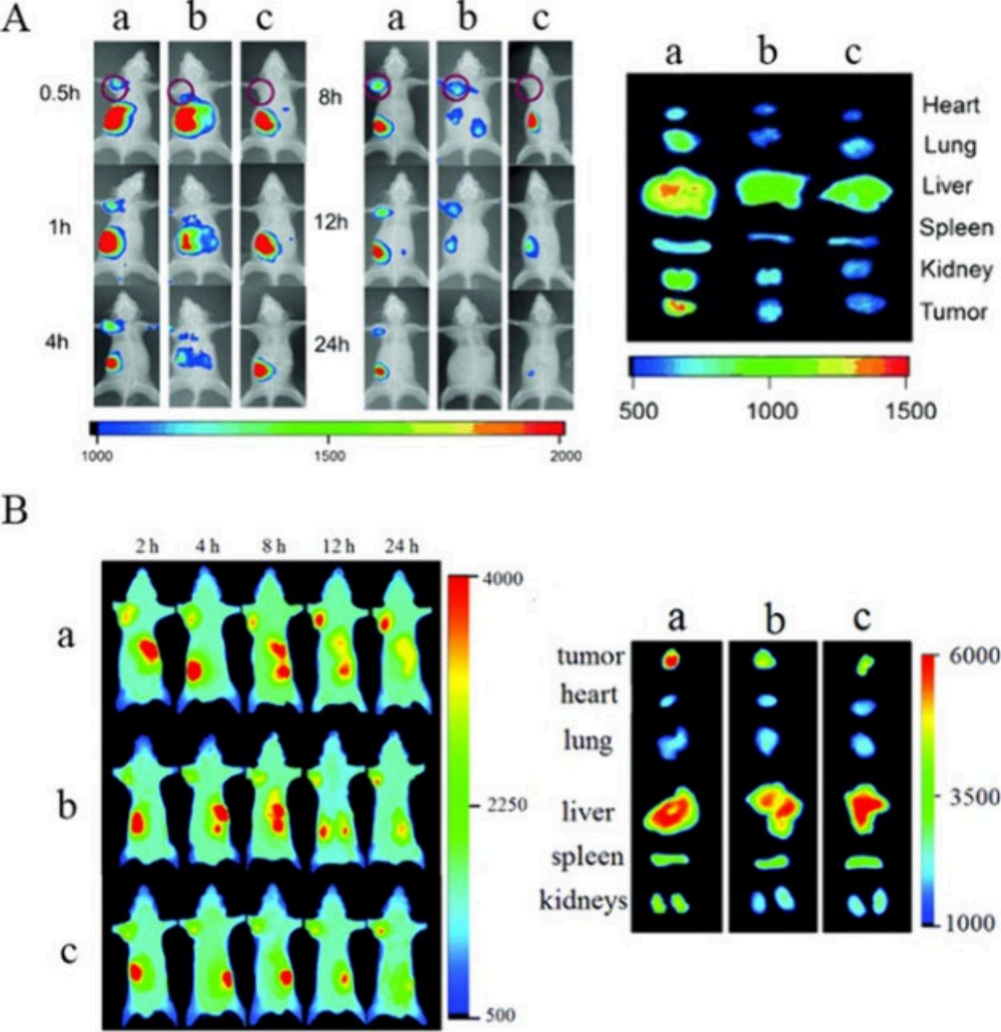
In a subcutaneous tumor-bearing mice model, NP distribution was observed at various time points *in vivo*, alongside fluorescence imaging of major organs and tumors *ex vivo* at 24 h postinjection. A) a. shows the conformational epitope of p32-imprinted NPs, b. conformational epitope of Lyp-1 (a peptide ligand binding to the N-terminal domain of p32) imprinted NPs, c. nonimprinted NPs.^48^ Reprinted with permission. Copyright 2015, Wiley-VCH.^48^ B) a. Illustrates the conformational epitope of FRα-imprinted NPs, b. scrambled epitope of FRα-imprinted NPs, c. nonimprinted NPs. Reprinted with permission. Copyright 2017, Royal Society of Chemistry.^49^

**Figure 3:**
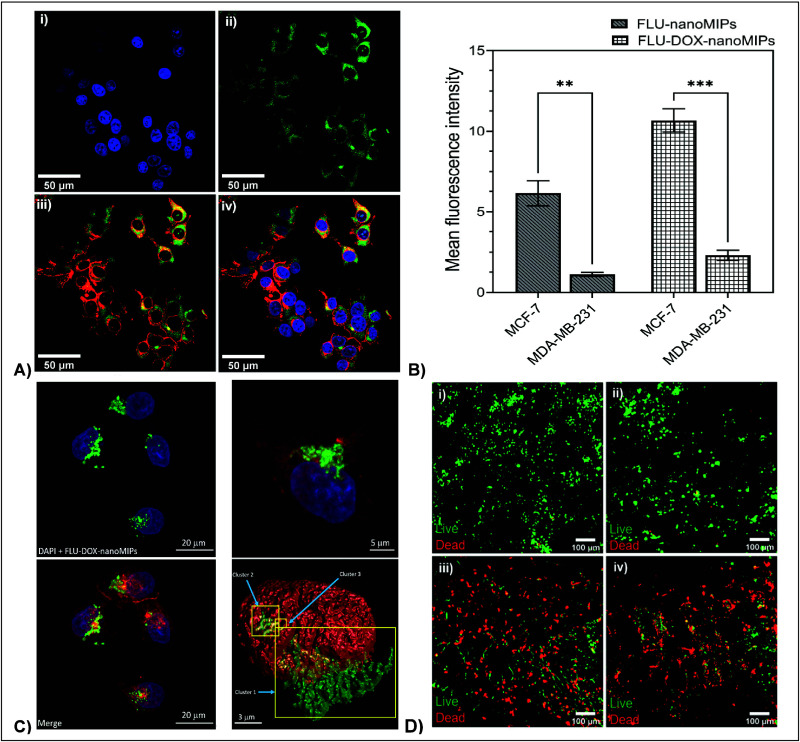
(A) Confocal images (after 1 h) of MCF-7 (ERα positive cell line) incubated with doxorubicin loaded and fluorescein tagged nanoMIPs (FLU-DOX-nanoMIPs): (i) DAPI, (ii) nanoMIPs with green fluorescence, and (iii) plasma membrane with red fluorescence (WGA antibody Alexa Fluor 594) with fluorescein tagged nanoMIPs, iv) merged. (B) The mean fluorescence intensity of FLU-nanoMIPs and FLU-DOX-nanoMIPs in MCF-7 cells and MDA-MB-231 cells (ERα negative cell line). (C) Translocation of nanoMIPs from membrane to nucleus; (D) 3D scaffolds of MCF-7 cell line showing live (green)/dead (red) staining: (i) control, (ii) fluorescein tagged nanoMIPs, (iii) DOX drug, iv) FLU-DOX-nanoMIPs (Reprinted from Singla et al., *Advanced Science*, p 2309976, 2023).^76^

p32, also known as the “Receptor of the globular head of C1q (gC1qR)” and the folate receptor-α (FR-α), has been found to be overexpressed in various cancer types.^50,51^ Zhang and colleagues used the conformational N-terminal epitope of the p32 receptor to synthesize nanoMIPs to recognize p32.^48^ The results showed that nanoMIPs were capable of specifically binding to both conformational and linear epitopes. In particular, nanoMIPs specifically bound to p32 positive cancer cells, thus leading to higher cellular uptake in these cells compared to control nonimprinted polymers. Consequently, the nanoMIPs showed an increased accumulation in p32-positive tumors in a mouse model (Figure 3B). Liu et al. synthesized nanoMIPs by imprinting a conformational epitope of FR-α.^49^ These nanoMIPs specifically targeted FRα-overexpressing HeLa cells without interference from the natural ligand, folate, both *in vitro* and *in vivo* (Figure 2B).

The reports on nanoMIPs for therapeutic use remain limited but follow-on work of Shea’s group focused on the development of nanoMIPs for anti-high-mobility group box 1 (HMGB1).^52^ HMGB1 is a multifunctional protein, and blocking of its functionality via binding of nanoMIPs to the receptor offers a therapeutic approach for treating ischemic injury. Upon injection of nanoMIPs (0–100 μg/mL) in rat brains, there was no increase in the levels of inflammatory cytokines (TNFα and IL-12) observed. However, the profile of these markers was only assessed over a short time frame and this is not sufficient to assess the true *in vivo* application of these materials, especially considering that degradation might occur due to the (loosely) cross-linked nature of the nanoMIPs.

A recent study evaluated uptake in tissue, biodistribution, and clearance of fluorescent nanoMIPs (100–200 nm) produced in aqueous systems in periods up to 168 h using trypsin as a model system. After oral and intravenous administration of nanoMIPs to rats, confocal microscopy revealed that the nanoMIPs were observed in all harvested tissue samples (in the brain, liver, spleen, and intestines when nanoMIPs were administered orally).^53^ The uptake of nanoMIPs in brain tissue is an exciting development which was not reported before, as most common small drugs are not able to penetrate the blood–brain barrier (BBB). Therefore, this establishes the potential for use of nanoMIPs to transport drugs for neurological conditions across the BBB, an area that has not been explored yet. However, at the same time, this may lead to potential issues related to the accumulation of these nanoMIPs in the brain. To improve the biocompatibility and intracellular uptake of imprinted polymeric and other polymeric nanoparticles, PEGylation (covalently attachment of polyethylene glycol), ionic liquid coating, and cell-penetrating peptides can be cross-linked.^54,55^

The study by Kassem et al. did highlight some concerns with regard to cytotoxicity caused by exposure to nanoMIPs *in vivo*, which could be due to the longer experimental time compared to previous studies.^53^ When trypsin nanoMIPs were administered intravenously to rats at lower concentrations (100 μg/L), a minimal effect on cells and tissue, such as infiltration of cells or presence of inflammatory cells, was observed. It is worth noting that even currently used clinical nanomaterials (e.g., monoclonal antibodies) have some level of nonspecific binding which leads to toxicity. However, a higher level of inflammatory biomarkers and more pronounced toxicity effects were observed in the case of oral administration of nanoMIPs or at higher dose (200 μg/L), underlining the importance of dose and administration mode. A caveat to the study is that sterilization techniques were not applied to the nanoMIPs, and therefore, it is not clear whether the observed effects were due to the material itself or potential residuals (solvents, initiators) originating from the synthesis. It has been well-established that autoclaving of nanoMIPs is possible without compromising affinity, and it should be considered whether this needs to be a standard practice before studying their behavior *in vivo*.^56^ Moreover, purification of product is also important to ensure that the cytotoxic response is not due to residues from the reaction.

A further key trepidation for the clinical application of nanoMIPs is that prolonged exposure to these materials can induce an immune response, whether positive or negative immune response remains to be evaluated, which can be dependent on the nanoMIP used. The size of the materials is crucially important to dictate the method of clearance; NPs with a size of <10–20 nm can escape the liver and spleen macrophages and would primarily be excreted via a renal pathway, which generally results in decreased toxicity. However, the majority of nanoMIPs are on the order of 50–150 nm (depending on the clinical application), where one would expect clearance by liver and spleen macrophages as reported in the literature,^57^ which is associated with longer exposure of nanoMIPs in the body. It must be noted that it depends on the intended clinical application of the nanoMIPs.

For drug delivery, NPs with a size of 50–200 nm are generally considered to be suitable candidates for drug delivery due to their high retention time, large capacity for therapeutic payloads, and enhanced permeability.^58,59^ However, this might be different for *in vivo* diagnostics or therapeutics. In addition, the overall charge and softness of the materials also has a significant impact on clearance besides the size. However, one option to overcome the buildup of nanoMIP is to include a switch that may allow degradation of the nanoMIP on payload delivery. This degradation process could break down the nanoMIP to smaller sizes which can then be cleared through the normal physiological pathways.

The immunogenicity of nanoMIPs is comparable to that of other polymeric nanoparticles, as their interaction with the immune system is influenced by their physicochemical properties.^60^ Nanomaterials including nanoMIPs with highly charged surfaces tend to associate with plasma proteins, making them more readily absorbed by phagocytic cells.^61^ Immunogenicity negatively impacts the use of nanoMIPs as drug carriers or *in vivo* diagnostic materials. This stimulation is undesirable and must be assessed before using these nanomaterials for such applications. One approach is to measure the surface marker expression of CD40 and CD86; their upregulation is indicative of the activation of antigen-presenting dendritic cells. This activation subsequently stimulates T cells and induces an immune response.^62^ An indirect method involves determining the levels of cytokines such as TNF-alpha and interleukins, which increase in response to an immune reaction. Canfarotta and colleagues screened the nanoMIPs for the levels of cytokines and chemokines (IL-1α, IL-1β, MCP-1, TNFα, and rKC) on macrophages. Results showed that there was no enhancement in cytokine levels except MCP-1, suggesting a low probability of these nanoMIPs inducing inflammatory and immunogenic responses. However, an increase in MCP-1 levels was observed, recommending further monitoring of neutrophil and monocyte activity^54^ with an *in vitro* comparative immunogenicity assessment (IVCIA) assay. While useful for risk ranking and candidate selection, the assay is limited by the absence of key *in vivo* factors, such as administration route, antigen-presenting cell processing, and interactions with other cell types and tissues.^63,64^ Although it can identify potential clinical immunogenicity, the assay cannot predict immunogenicity rates in clinical settings, which require a multidose clinical assessment. Immunogenicity is a crucial parameter for clinical application of nanoMIPs but there are no comprehensive immunogenicity studies reported in the literature yet.

### 3.2 Model Systems

Traditionally, animal models have been used to predict the *in vivo* behavior of NPs in clinical application. However, it has been well-established that these animal models are not always able to accurately capture the complexities of the human environment. Mice have remained the traditional experimental model in the field of biomedical research but have significantly different dietary requirements, lifestyle and microbiomes compared to humans. Alternative animal models, in particular those (e.g., fertilized hen-eggs, zebrafish embryos) that have a less severe impact on animal welfare, should be considered. Cecchini et al. coupled nanoMIPs with quantum dots (QD) to employ them for imaging of vascular endothelial growth factor, which is overexpressed in certain cancer types.^65^ To evaluate potential toxic effects, nanoMIPs were injected into the yolks of zebrafish embryos. It was shown that there was no significant difference (*n* = 40, *p* > 0.5, chi-squared test) between embryos injected with nanoMIPs and relevant controls.

There is a growing interest in sophisticated animal-free technologies to predict the *in vivo* behavior of NPs. This is fueled by the European policy, Directive 2010/63/EU, which prohibits the use of animals where alternative models exist.^66^ Moreover, the Food Drug Authority Modernization Act 2.0 that was approved in 2022 allows for drug makers to collect initial safety and efficacy data using tools such as organ on chips and 3D tissue constructs instead of live animals. *In vitro* tissue constructs/models are three-dimensional structures that can capture features that are present in actual tissues and that are important for the tissue response to a therapy. Examples of those features are the following: (i) cellular features and cellular complexity (especially in 3D models consisting of multiple cell types such as diseased cells and healthy surrounding cells which can interact with the diseased populations), (ii) biochemical features and more specifically Extracellular Matrix Proteins (ECM), (iii) biomechanical features (e.g., stiffness). Furthermore, immersing 3D constructs into bioreactors enables mimicking the interstitial flow. For the development/generation of 3D tissue models, commonly used biomaterials (synthetic or natural) are employed to generate 3D structures of various structural biochemical configurations (hydrogels or polymeric scaffolds). There are also models using a combination of materials, e.g. a synthetic polymer combined with a natural polymer. Inclusion of spatial complexity in the models by mapping different cell areas (e.g., fibrosis) provides an additional advantage toward better biomimicry *in vitro*.^67−75^ Singla et al. employed one such model,^76^ which is a polyurethane based scaffold, surface modified with ECM proteins for ECM mimicry and loaded with breast cancer cells, to evaluate the action of nanoMIPs. More specifically, the developed nanoMIPs targeted a linear epitope of estrogen receptor alpha (ERα), and the nanoMIPs were loaded with drug doxorubicin to achieve specific drug delivery toward ERα+ positive breast cancer cells (Figure 3).^76^

There has been one report in the literature to date on double imprinting using a membrane receptor, but this study was the first to report on nuclear drug delivery using this innovative approach that uses two targets of distinctly different size and functionality (a protein and a small drug compound).^76^ High efficacy of the nanoMIPs was shown both in the 2D models and in preliminary studies using these complex 3D models. Future studies will focus on incorporating patient cell lines to enable a true precision medicine approach for testing of novel drugs and evaluating of toxicity.

While promising, complications might arise when using this system for the delivery of hydrophobic drugs that are not water-soluble. Moreover, drug delivery for this method relies on diffusion and thus can be hard to control, rather than having systems that are triggered by, for instance, difference in pH, redox environment, and temperature. Therefore, it might be worth considering the payload to the imprinted nanoMIPs with cleavable linkers, such as is done for antibody-drug conjugates (ADCs).

## 4 Future Perspective

It has been shown that nanoMIPs can rival the affinity of commercial antibodies while offering the advantages of low-cost, robustness, versatility, and being an animal-free technology. Therefore, there are immediate applications for these materials as antibody replacements, such as in diagnostic assays and sensors. However, the true strengths of nanoMIPs lie in their “soluble” format and their potential for multiple functionalities beyond just recognition, which opens up the opportunity to use these materials for drug delivery, therapeutics, and theragnostics. To reach their full potential, we have discussed in this Perspective which advances are needed in the development and in manufacturing and have presented a thorough investigation of the *in vivo* behavior of nanoMIPs. In particular, we predict that automating MIP manufacturing, which is possible with computational approaches/AI and innovative reactor designs, in addition to high throughput screening to predict their clinical behavior, will be necessary to achieve breakthroughs in this field. Biodegradable imprinted polymers^77^ would show promise in this regard, since they naturally degrade into smaller parts that can be cleared without accumulating in the tissue. Further avenues of research are likely to be around the combination of nanoMIPs with cleavable linkers and cell-penetrating peptides (CPPs) for healthcare applications. The future perspectives of using CPPs applied to nanoMIPs for personalized medicine, drug delivery, and vaccine development are highly promising and include several possible key advancements such as enhanced delivery efficiency, personalized medicine approaches, and overcoming biological barriers to expand the range of diseases that can be targeted.

## References

[ref1] Vasapollo G.; Sole R. D.; Mergola L.; Lazzoi M. R.; Scardino A.; Scorrano S.; Mele G. Molecularly imprinted polymers: present and future prospective. Int. J. Mol. Sci. 2011, 12 (9), 5908–5945. 10.3390/ijms12095908. 22016636 PMC3189760

[ref2] Resmini M. Molecularly imprinted polymers as biomimetic catalysts. Anal Bioanal Chem. 2012, 402, 3021–3026. 10.1007/s00216-011-5671-2. 22245980

[ref3] Staiano M.; Pennacchio A.; Varriale A.; Capo A.; Majoli A.; Capacchione C.; D’Auria S. Enzymes as sensors. Methods Enzymol. 2017, 589, 115–131. 10.1016/bs.mie.2017.01.015. 28336061

[ref4] Ertürk G.; Mattiasson B. Molecular imprinting techniques used for the preparation of biosensors. Sensors 2017, 17 (2), 288 10.3390/s17020288. 28165419 PMC5335940

[ref5] Lowdon J. W.; Diliën H.; Singla P.; Peeters M.; Cleij T. J.; van Grinsven B.; Eersels K. MIPs for commercial application in low-cost sensors and assays-An overview of the current status quo. Sens. Actuators B Chem. 2020, 325, 128973 10.1016/j.snb.2020.128973. 33012991 PMC7525251

[ref6] Piletsky S.; Canfarotta F.; Poma A.; Bossi A. M.; Piletsky S. Molecularly imprinted polymers for cell recognition. Trends Biotechnol. 2020, 38 (4), 368–387. 10.1016/j.tibtech.2019.10.002. 31677857

[ref7] Pilvenyte G.; Ratautaite V.; Boguzaite R.; Ramanavicius A.; Viter R.; Ramanavicius S. Molecularly imprinted polymers for the determination of cancer biomarkers. Int. J. Mol. Sci. 2023, 24 (4), 4105 10.3390/ijms24044105. 36835517 PMC9961774

[ref8] Wang L.; Pagett M.; Zhang W. Molecularly imprinted polymer (MIP) based electrochemical sensors and their recent advances in health applications. Sens. Actuators Rep. 2023, 5, 100153 10.1016/j.snr.2023.100153.

[ref9] Çimen D.; Bereli N.; Günaydın S.; Denizli A. Molecular imprinted nanoparticle assisted surface plasmon resonance biosensors for detection of thrombin. Talanta 2022, 246, 123484 10.1016/j.talanta.2022.123484. 35462248

[ref10] Gray A.; Bradbury A. R.; Knappik A.; Plückthun A.; Borrebaeck C. A.; Dübel S. Animal-free alternatives and the antibody iceberg. Nat. Biotechnol. 2020, 38 (11), 1234–1239. 10.1038/s41587-020-0687-9. 33046876

[ref11] Polyakov M. Adsorption properties and structure of silica gel. Zhur Fiz Khim 1931, 2, 799–805.

[ref12] Dickey F. H. The preparation of specific adsorbents. Proc. Natl. Acad. Sci. U.S.A. 1949, 35 (5), 227–229. 10.1073/pnas.35.5.227. 16578311 PMC1063007

[ref13] Takagishi T.; Klotz I. M. Macromolecule-small molecule interactions; introduction of additional binding sites in polyethyleneimine by disulfide cross-linkages. Biopolymers 1972, 11 (2), 483–491. 10.1002/bip.1972.360110213. 5016558

[ref14] Wulff G.; Sarhan A. Über die Anwendung von enzymanalog gebauten Polymeren zur Racemattrennung. Angew. Chem. 1972, 84 (8), 364–364. 10.1002/ange.19720840838.

[ref15] Sellergren B.; Lepistoe M.; Mosbach K. Highly enantioselective and substrate-selective polymers obtained by molecular imprinting utilizing noncovalent interactions. NMR and chromatographic studies on the nature of recognition. J. Am. Chem. Soc. 1988, 110 (17), 5853–5860. 10.1021/ja00225a041.

[ref16] Vlatakis G.; Andersson L. I.; Müller R.; Mosbach K. Drug assay using antibody mimics made by molecular imprinting. Nature 1993, 361 (6413), 645–647. 10.1038/361645a0. 8437624

[ref17] Refaat D.; Aggour M. G.; Farghali A. A.; Mahajan R.; Wiklander J. G.; Nicholls I. A.; Piletsky S. A. Strategies for molecular imprinting and the evolution of MIP nanoparticles as plastic antibodies—Synthesis and applications. Int. J. Mol. Sci. 2019, 20 (24), 6304 10.3390/ijms20246304. 31847152 PMC6940816

[ref18] Tchekwagep P. M. S.; Crapnell R. D.; Banks C. E.; Betlem K.; Rinner U.; Canfarotta F.; Lowdon J. W.; Eersels K.; van Grinsven B.; Peeters M. A critical review on the use of molecular imprinting for trace heavy metal and micropollutant detection. Chemosensors 2022, 10 (8), 296 10.3390/chemosensors10080296.

[ref19] Canfarotta F.; Czulak J.; Betlem K.; Sachdeva A.; Eersels K.; Van Grinsven B.; Cleij T.; Peeters M. A novel thermal detection method based on molecularly imprinted nanoparticles as recognition elements. Nanoscale 2018, 10 (4), 2081–2089. 10.1039/C7NR07785H. 29323388

[ref20] Chianella I.; Guerreiro A.; Moczko E.; Caygill J. S.; Piletska E. V.; De Vargas Sansalvador I. M. P.; Whitcombe M. J.; Piletsky S. A. Direct Replacement of Antibodies with Molecularly Imprinted Polymer Nanoparticles in ELISA- Development of a Novel Assay for Vancomycin. Anal. Chem. 2013, 85 (17), 8462–8468. 10.1021/ac402102j. 23947402 PMC4720989

[ref21] Moczko E.; Díaz R.; Rivas B.; García C.; Pereira E.; Piletsky S.; Cáceres C. Molecularly imprinted nanoparticles assay (MINA) in pseudo ELISA: an alternative to detect and quantify octopamine in water and human urine samples. Polymers 2019, 11 (9), 1497 10.3390/polym11091497. 31540212 PMC6780943

[ref22] McClements J.; Bar L.; Singla P.; Canfarotta F.; Thomson A.; Czulak J.; Johnson R.; Crapnell R.; Banks C.; Payne B. Molecularly imprinted polymer nanoparticles enable rapid, reliable, and robust point-of-care thermal detection of SARS-CoV-2. ACS Sens. 2022, 7 (4), 1122–1131. 10.1021/acssensors.2c00100. 35416035 PMC9016778

[ref23] Poma A.; Guerreiro A.; Whitcombe M. J.; Piletska E. V.; Turner A. P.; Piletsky S. A. Solid-phase synthesis of molecularly imprinted polymer nanoparticles with a reusable template-“plastic antibodies. Adv. Funct. Mater. 2013, 23 (22), 2821–2827. 10.1002/adfm.201202397. 26869870 PMC4746745

[ref24] Canfarotta F.; Poma A.; Guerreiro A.; Piletsky S. Solid-phase synthesis of molecularly imprinted nanoparticles. Nat. Protoc. 2016, 11, 443 10.1038/nprot.2016.030. 26866789

[ref25] Singla P.; Kaur S.; Jamieson O.; Dann A.; Garg S.; Mahon C.; Crapnell R. D.; Banks C. E.; Kaur I.; Peeters M. Electrochemical and thermal detection of allergenic substance lysozyme with molecularly imprinted nanoparticles. Anal Bioanal Chem. 2023, 415 (18), 4467–4478. 10.1007/s00216-023-04638-2. 36905407 PMC10329058

[ref26] Di Masi S.; Costa M.; Canfarotta F.; Guerreiro A.; Hartley A.; Piletsky S. A.; Malitesta C. An impedimetric sensor based on molecularly imprinted nanoparticles for the determination of trypsin in artificial matrices-towards point-of-care diagnostics. Anal. Methods 2024, 16 (5), 742–750. 10.1039/D3AY01762A. 38224108

[ref27] Teixeira S. P.; Reis R. L.; Peppas N. A.; Gomes M. E.; Domingues R. M. Epitope-imprinted polymers: Design principles of synthetic binding partners for natural biomacromolecules. Sci. Adv. 2021, 7 (44), eabi9884 10.1126/sciadv.abi9884. 34714673 PMC8555893

[ref28] Truta F.; Cruz A. G.; Tertis M.; Zaleski C.; Adamu G.; Allcock N. S.; Suciu M.; Ştefan M.-G.; Kiss B.; Piletska E. NanoMIPs-based electrochemical sensors for selective detection of amphetamine. Microchem. J. 2023, 191, 108821 10.1016/j.microc.2023.108821.

[ref29] Nicholls I. A.; Golker K.; Olsson G. D.; Suriyanarayanan S.; Wiklander J. G. The use of computational methods for the development of molecularly imprinted polymers. Polymers 2021, 13 (17), 2841 10.3390/polym13172841. 34502881 PMC8434026

[ref30] Lowdon J. W.; Ishikura H.; Kvernenes M. K.; Caldara M.; Cleij T. J.; van Grinsven B.; Eersels K.; Diliën H. Identifying Potential Machine Learning Algorithms for the Simulation of Binding Affinities to Molecularly Imprinted Polymers. Computation 2021, 9 (10), 103 10.3390/computation9100103.

[ref31] Karasu T.; Çalışır F.; Pişkin S.; Özgür E.; Armutcu C.; Çorman M. E.; Uzun L. An intriguing future is approaching: Artificial intelligence meets molecularly imprinted polymers. JPBA Open 2024, 100041.

[ref32] Zhao S.; Liu W.; Song D. Rapid detection and prediction model establishment of propachlor residues in food assisted by machine learning. J. Food Meas. Charact. 2023, 17 (6), 5972–5979. 10.1007/s11694-023-02084-3.

[ref33] Herrera-Chacon A.; González-Calabuig A.; Campos I.; del Valle M. Bioelectronic tongue using MIP sensors for the resolution of volatile phenolic compounds. Sens. Actuators B Chem. 2018, 258, 665–671. 10.1016/j.snb.2017.11.136.

[ref34] Dykstra G.; Reynolds B.; Smith R.; Zhou K.; Liu Y. Electropolymerized molecularly imprinted polymer synthesis guided by an integrated data-driven framework for cortisol detection. ACS Appl. Mater. Interfaces. 2022, 14 (22), 25972–25983. 10.1021/acsami.2c02474. 35536156

[ref35] Yarahmadi B.; Hashemianzadeh S. M.; Milani Hosseini S. M.-R. Machine-learning-based predictions of imprinting quality using ensemble and non-linear regression algorithms. Sci. Rep. 2023, 13 (1), 12111 10.1038/s41598-023-39374-1. 37495673 PMC10372080

[ref36] Zourob M.; Mohr S.; Mayes A. G.; Macaskill A.; Pérez-Moral N.; Fielden P. R.; Goddard N. J. A micro-reactor for preparing uniform molecularly imprinted polymer beads. LOC 2006, 6 (2), 296–301. 10.1039/b513195b. 16450041

[ref37] Jafari V. F.; Mossayebi Z.; Allison-Logan S.; Shabani S.; Qiao G. G. The Power of Automation in Polymer Chemistry: Precision Synthesis of Multiblock Copolymers with Block Sequence Control. Chem.—Eur. J. 2023, 29 (53), e202301767 10.1002/chem.202301767. 37401148

[ref38] Wilding C. Y.; Knox S. T.; Bourne R. A.; Warren N. J. Development and Experimental Validation of a Dispersity Model for In Silico RAFT Polymerization. Macromolecules 2023, 56 (4), 1581–1591. 10.1021/acs.macromol.2c01798. 36874531 PMC9979647

[ref39] Kershaw O. J.; Clayton A. D.; Manson J. A.; Barthelme A.; Pavey J.; Peach P.; Mustakis J.; Howard R. M.; Chamberlain T. W.; Warren N. J. Machine learning directed multi-objective optimization of mixed variable chemical systems. Chem. Eng. J. 2023, 451, 138443 10.1016/j.cej.2022.138443.

[ref40] Taylor C. J.; Pomberger A.; Felton K. C.; Grainger R.; Barecka M.; Chamberlain T. W.; Bourne R. A.; Johnson C. N.; Lapkin A. A. A brief introduction to chemical reaction optimization. Chem. Rev. 2023, 123 (6), 3089–3126. 10.1021/acs.chemrev.2c00798. 36820880 PMC10037254

[ref41] Pittaway P. M.; Ghasemi G.; Knox S. T.; Cayre O. J.; Kapur N.; Warren N. J. Continuous synthesis of block copolymer nanoparticles via telescoped RAFT solution and dispersion polymerisation in a miniature CSTR cascade. React. Chem. Eng. 2023, 8 (3), 707–717. 10.1039/D2RE00475E.

[ref42] O’Mahony J. M. Designing molecularly imprinted polymers for the analysis of the components of complex matrices. Dublin City University, 2004.

[ref43] Sullivan M. V.; Nanalal S.; Dean B. E.; Turner N. W. Molecularly imprinted polymer hydrogel sheets with metalloporphyrin-incorporated molecular recognition sites for protein capture. Talanta 2024, 266, 125083 10.1016/j.talanta.2023.125083. 37598443

[ref44] Hudson A. D.; Jamieson O.; Crapnell R. D.; Rurack K.; Soares T. C.; Mecozzi F.; Laude A.; Gruber J.; Novakovic K.; Peeters M. Dual detection of nafcillin using a molecularly imprinted polymer-based platform coupled to thermal and fluorescence read-out. Mater. Adv. 2021, 2 (15), 5105–5115. 10.1039/D1MA00192B.

[ref45] Vert M.; Doi Y.; Hellwich K.-H.; Hess M.; Hodge P.; Kubisa P.; Rinaudo M.; Schué F. Terminology for biorelated polymers and applications (IUPAC Recommendations 2012). Pure Appl. Chem. 2012, 84 (2), 377–410. 10.1351/PAC-REC-10-12-04.

[ref46] Mier A.; Nestora S.; Medina Rangel P. X.; Rossez Y.; Haupt K.; Tse Sum Bui B. Cytocompatibility of molecularly imprinted polymers for deodorants: Evaluation on human keratinocytes and axillary-hosted bacteria. ACS Appl. Bio Mater. 2019, 2 (8), 3439–3447. 10.1021/acsabm.9b00388. 35030732

[ref47] Hoshino Y.; Koide H.; Urakami T.; Kanazawa H.; Kodama T.; Oku N.; Shea K. J. Recognition, neutralization, and clearance of target peptides in the bloodstream of living mice by molecularly imprinted polymer nanoparticles: a plastic antibody. J. Am. Chem. Soc. 2010, 132 (19), 6644–6645. 10.1021/ja102148f. 20420394 PMC2874824

[ref48] Zhang Y.; Deng C.; Liu S.; Wu J.; Chen Z.; Li C.; Lu W. Active targeting of tumors through conformational epitope imprinting. Angew. Chem., Int. Ed. 2015, 54 (17), 5157–5160. 10.1002/anie.201412114. 25727886

[ref49] Liu S.; Bi Q.; Long Y.; Li Z.; Bhattacharyya S.; Li C. Inducible epitope imprinting:‘generating’the required binding site in membrane receptors for targeted drug delivery. Nanoscale 2017, 9 (17), 5394–5397. 10.1039/C6NR09449J. 28422195

[ref50] Egusquiza-Alvarez C. A.; Castañeda-Patlán M. C.; Albarran-Gutierrez S.; Gonzalez-Aguilar H.; Moreno-Londoño A. P.; Maldonado V.; Melendez-Zajgla J.; Robles-Flores M. Overexpression of multifunctional protein p32 promotes a malignant phenotype in colorectal cancer cells. Front. Oncol. 2021, 11, 642940 10.3389/fonc.2021.642940. 34136383 PMC8201776

[ref51] Bellotti E.; Cascone M. G.; Barbani N.; Rossin D.; Rastaldo R.; Giachino C.; Cristallini C. Targeting cancer cells overexpressing folate receptors with new terpolymer-based nanocapsules: Toward a novel targeted dna delivery system for cancer therapy. Biomedicines 2021, 9 (9), 1275 10.3390/biomedicines9091275. 34572461 PMC8471118

[ref52] Koide H.; Kiyokawa C.; Okishima A.; Saito K.; Yoshimatsu K.; Fukuta T.; Hoshino Y.; Asai T.; Nishimura Y.; Miura Y.; Oku N.; Shea K. J. Design of an Anti-HMGB1 synthetic antibody for in vivo ischemic/reperfusion injury therapy. J. Am. Chem. Soc. 2023, 145 (42), 23143–23151. 10.1021/jacs.3c06799. 37844138 PMC10603801

[ref53] Kassem S.; Piletsky S. S.; Yesilkaya H.; Gazioglu O.; Habtom M.; Canfarotta F.; Piletska E.; Spivey A. C.; Aboagye E. O.; Piletsky S. A. Assessing the in vivo biocompatibility of molecularly imprinted polymer nanoparticles. Polymers 2022, 14 (21), 4582 10.3390/polym14214582. 36365575 PMC9655879

[ref54] Canfarotta F.; Waters A.; Sadler R.; McGill P.; Guerreiro A.; Papkovsky D.; Haupt K.; Piletsky S. Biocompatibility and internalization of molecularly imprinted nanoparticles. Nano Res. 2016, 9, 3463–3477. 10.1007/s12274-016-1222-7.

[ref55] Singh G.; Dasanayake G. S.; Chism C. M.; Vashisth P.; Kaur A.; Misra S. K.; Sharp J. S.; Tanner E. E. Good’s buffer based highly biocompatible ionic liquid modified PLGA nanoparticles for the selective uptake in cancer cells. Mater. Chem. Front. 2023, 7 (24), 6213–6228. 10.1039/D3QM00787A. 38204762 PMC10776129

[ref56] McClements J.; Seumo Tchekwagep P. M.; Vilela Strapazon A. L.; Canfarotta F.; Thomson A.; Czulak J.; Johnson R. E.; Novakovic K.; Losada-Perez P.; Zaman A. Immobilization of molecularly imprinted polymer nanoparticles onto surfaces using different strategies: evaluating the influence of the functionalized interface on the performance of a thermal assay for the detection of the cardiac biomarker troponin I. ACS Appl. Mater. Interfaces 2021, 13 (24), 27868–27879. 10.1021/acsami.1c05566. 34110781

[ref57] Ngo W.; Ahmed S.; Blackadar C.; Bussin B.; Ji Q.; Mladjenovic S. M.; Sepahi Z.; Chan W. C. Why nanoparticles prefer liver macrophage cell uptake in vivo. Adv. Drug Delivery Rev. 2022, 185, 114238 10.1016/j.addr.2022.114238. 35367524

[ref58] Fang J.; Nakamura H.; Maeda H. The EPR effect: unique features of tumor blood vessels for drug delivery, factors involved, and limitations and augmentation of the effect. Adv. Drug Delivery Rev. 2011, 63 (3), 136–151. 10.1016/j.addr.2010.04.009. 20441782

[ref59] Nel A.; Ruoslahti E.; Meng H. New insights into “permeability” as in the enhanced permeability and retention effect of cancer nanotherapeutics. ACS 2017, 11 (10), 9567–9569. 10.1021/acsnano.7b07214. 29065443

[ref60] Dobrovolskaia M. A.; McNeil S. E. Immunological properties of engineered nanomaterials. Nat. Nanotechnol. 2007, 2 (8), 469–478. 10.1038/nnano.2007.223. 18654343

[ref61] Owens D. E. III; Peppas N. A. Opsonization, biodistribution, and pharmacokinetics of polymeric nanoparticles. Int. J. Pharm. 2006, 307 (1), 93–102. 10.1016/j.ijpharm.2005.10.010. 16303268

[ref62] Kaneko K.; Miyaji E. N.; Gonçalves V. M.; Ferreira D. M.; Solórzano C.; MacLoughlin R.; Saleem I. Evaluation of polymer choice on immunogenicity of chitosan coated PLGA NPs with surface-adsorbed pneumococcal protein antigen PspA4Pro. Int. J. Pharm. 2021, 599, 120407 10.1016/j.ijpharm.2021.120407. 33675930 PMC8188518

[ref63] Moussa E. M.; Panchal J. P.; Moorthy B. S.; Blum J. S.; Joubert M. K.; Narhi L. O.; Topp E. M. Immunogenicity of therapeutic protein aggregates. J. Pharm. Sci. 2016, 105 (2), 417–430. 10.1016/j.xphs.2015.11.002. 26869409

[ref64] Jawa V.; Cousens L. P.; Awwad M.; Wakshull E.; Kropshofer H.; De Groot A. S. T-cell dependent immunogenicity of protein therapeutics: preclinical assessment and mitigation. Clin. Immunol. 2013, 149 (3), 534–555. 10.1016/j.clim.2013.09.006. 24263283

[ref65] Cecchini A.; Raffa V.; Canfarotta F.; Signore G.; Piletsky S.; MacDonald M. P.; Cuschieri A. In vivo recognition of human vascular endothelial growth factor by molecularly imprinted polymers. Nano Lett. 2017, 17 (4), 2307–2312. 10.1021/acs.nanolett.6b05052. 28350162

[ref66] Directive E. 63/EU of the European Parliament and of the Council of 22 September 2010 on the protection of animals used for scientific purposes. Off. J. Eur. Union 2010, 276, 33–79.

[ref67] Gupta P.; Bermejo-Rodriguez C.; Kocher H.; Pérez-Mancera P. A.; Velliou E. G. Chemotherapy Assessment in Advanced Multicellular 3D Models of Pancreatic Cancer: Unravelling the Importance of Spatiotemporal Mimicry of the Tumor Microenvironment. Adv. Biol. 2024, 8, 2300580 10.1002/adbi.202300580. 38327154

[ref68] Gupta P.; Pérez-Mancera P. A.; Kocher H.; Nisbet A.; Schettino G.; Velliou E. G. A novel scaffold-based hybrid multicellular model for pancreatic ductal adenocarcinoma—toward a better mimicry of the in vivo tumor microenvironment. Front. Bioeng. Biotechnol. 2020, 8, 290 10.3389/fbioe.2020.00290. 32391339 PMC7193232

[ref69] Gupta P.; Velliou E. G. A Step-by-Step Methodological Guide for Developing Zonal Multicellular Scaffold-Based Pancreatic Cancer Models. In Cancer Cell Culture: Methods and Protocols; Springer, 2023; pp 221–229. 10.1007/978-1-0716-3056-3_13 37202622

[ref70] Wishart G.; Gupta P.; Schettino G.; Nisbet A.; Velliou E. 3d tissue models as tools for radiotherapy screening for pancreatic cancer. Br J. Radiol. 2021, 94 (1120), 20201397 10.1259/bjr.20201397. 33684308 PMC8010544

[ref71] Wishart G.; Gupta P.; Nisbet A.; Schettino G.; Velliou E. On the evaluation of a novel hypoxic 3D pancreatic cancer model as a tool for radiotherapy treatment screening. Cancers 2021, 13 (23), 6080 10.3390/cancers13236080. 34885188 PMC8657010

[ref72] Wishart G.; Gupta P.; Nisbet A.; Velliou E.; Schettino G. Enhanced effect of X-rays in the presence of a static magnetic field within a 3D pancreatic cancer model. Br J. Radiol. 2023, 96 (1143), 20220832 10.1259/bjr.20220832. 36475863 PMC9975369

[ref73] Totti S.; Allenby M. C.; Dos Santos S. B.; Mantalaris A.; Velliou E. G. A 3D bioinspired highly porous polymeric scaffolding system for in vitro simulation of pancreatic ductal adenocarcinoma. RSC Adv. 2018, 8 (37), 20928–20940. 10.1039/C8RA02633E. 35542351 PMC9080900

[ref74] Totti S.; Vernardis S. I.; Meira L.; Pérez-Mancera P. A.; Costello E.; Greenhalf W.; Palmer D.; Neoptolemos J.; Mantalaris A.; Velliou E. G. Designing a bio-inspired biomimetic in vitro system for the optimization of ex vivo studies of pancreatic cancer. Drug Discovery Today 2017, 22 (4), 690–701. 10.1016/j.drudis.2017.01.012. 28153670

[ref75] Totti S.; Ng K. W.; Dale L.; Lian G.; Chen T.; Velliou E. G. A novel versatile animal-free 3D tool for rapid low-cost assessment of immunodiagnostic microneedles. Sens. Actuators B Chem. 2019, 296, 126652 10.1016/j.snb.2019.126652.

[ref76] Singla P.; Broughton T.; Sullivan M. V.; Garg S.; Berlinguer-Palmini R.; Gupta P.; Smith K. J.; Gardner B.; Canfarotta F.; Turner N. W. Double Imprinted Nanoparticles for Sequential Membrane-to-Nuclear Drug Delivery. Adv. Sci. 2024, 2309976 10.1002/advs.202309976. PMC11423068 38973256

[ref77] Nguyen M.-H.; Onken A.; Sündermann J.; Shamsuyeva M.; Singla P.; Depuydt T.; Peeters M.; Wagner P.; Bethmann K.; Körner J. Electrochemical Degradation of Molecularly Imprinted Polymers for Future Applications of Inflammation Sensing in Cochlear Implants. ACS Omega 2024, 9, 25223 10.1021/acsomega.4c02906. 38882102 PMC11170751

